# A tale of two trials: The impact of 5α-reductase inhibition on prostate cancer (Review)

**DOI:** 10.3892/ol.2014.2388

**Published:** 2014-07-28

**Authors:** JOHN M. LACY, NATASHA KYPRIANOU

**Affiliations:** 1 Department of Urology, University of Kentucky College of Medicine, Lexington, KY 40536-0293, USA; 2 Department of Molecular and Cellular Biochemistry, University of Kentucky College of Medicine, Lexington, KY 40536-0293, USA

**Keywords:** prostate cancer, clinical trials, chemoprevention, 5α-reductase inhibitors, REDUCE, PCPT

## Abstract

The use of 5α-reductase inhibitors (5α-RIs) as prostate cancer chemoprevention agents is controversial. Two large randomized trials, the Prostate Cancer Prevention Trial (PCPT) and the Reduction by Dutasteride of Prostate Cancer Events (REDUCE) Trial, have both shown a decreased incidence of prostate cancer in patients administered with 5α-RIs. Both studies showed, however, an increased risk of higher-grade prostate cancer. Numerous studies have since analyzed the inherent biases in these landmark studies and have used mathematical modeling to estimate the true incidence of prostate cancer and the risk for high-grade prostate cancer in patients undergoing 5α-RI treatment. All primary publications associated with the PCPT and REDUCE studies were reviewed in detail. Pertinent references from the above publications were assessed and a literature search of all published articles associated with PCPT, REDUCE or 5α-RIs as chemopreventative agents through October 2013 was performed using Pubmed/Medline. PCPT and REDUCE both showed a significant decrease in the incidence of prostate cancer following the administration of 5α-reductase inhibitor, as compared with placebo, suggesting that 5α-RIs may be effective agents for prostate cancer chemoprevention. Inherent biases in the design of these two studies may have caused an artificial increase in the number of high-grade cancers reported. Mathematical models, that integrated data from these trials, revealed neither an increased nor decreased risk of high-grade disease when taking these biases into consideration. Moderately strong evidence exists that 5α-RIs may reduce the risk of prostate cancer. PCPT and REDUCE showed a decreased prevalence of prostate cancer in patients taking 5α-RIs. Urologists should have a working knowledge of these studies and discuss with patients the risks and benefits of 5α-RI treatment. Further studies to evaluate the cost-effectiveness of chemoprevention with 5α-RIs and appropriate patient selection are warranted.

## 1. Introduction

Prostate cancer is the second most common cancer among men, with 206,640 diagnoses and 28,088 prostate cancer-associated deaths reported in 2009 (1). The decreased mortality rate of prostate cancer in the prostate-specific antigen test (PSA) era is well-documented (2). This is widely credited due to the possibility for earlier diagnosis of the disease, which enables treatment while the cancer remains localized in the prostate. With an increased rate in diagnoses of prostate cancer came concerns of over-treatment of clinically insignificant tumors and significant treatment side effects that have put prostate cancer into the national public health spotlight (3). These concerns culminated in May 2012 when the United States Preventative Task Force recommended against the use of PSA as a screening test for prostate cancer (3).

Prostate cancer has been considered to be an ideal tumor for chemoprevention due to its multi-step molecular pathway, prolonged latent phase and increased incidence with age (4). Several trials have pursued agents that could decrease the incidence and/or progression of prostate cancer (4). The Selenium and Vitamin E Cancer Prevention Trial investigated the impact of vitamin E and selenium alone and in combination on prostate cancer, and produced unexpected negative results. There was a reported increase in prostate cancer incidence in the group receiving vitamin E, while no significant effect was detected in the other groups (5). Recognizing the tumor-enhancing effects of an agent that was previously reported by a wealth of experimental basic findings as being chemopreventive, severely challenged the translational significance in a clinical setting (6). The Alpha-Tocopherol, Beta Carotene Prevention Trial showed initial promising results for prostate cancer prevention, but these results did not generate significance as the study progressed (7). Murine studies of the selective estrogen receptor modulator toremifene revealed its potential for delaying prostate cancer progression, but studies in human subjects have failed to deliver positive results (8). Other common medications, such as statins and nonsteroidal anti-inflammatory drugs, have also been evaluated in numerous studies with controversial results (6,12).

5α-reductase inhibitors (5α-RIs) have been among the most controversial candidates in the search for chemopreventative agents for prostate cancer; 5α-reductase converts testosterone to the intracellular active dihydrotestosterone, with a high affinity for the androgen receptor (13). Inhibitors of 5α-reductase activity are clinically used in the treatment of benign prostatic hyperplasia, through their ability to decrease the volume of the prostate gland in aging men. There are two isoenzymes of 5α-reductase: Type 1, which is expressed in the liver and skin, and type 2 that is expressed in the prostate (14). Finasteride (Proscar^®^) inhibits only the type 2 isoenzyme, but dutasteride (Avodart^®^) inhibits both the type 1 and type 2 isoenzymes. While the fundamentally significant contribution of androgens to prostate cancer etiology and progression had long been the focus of intense molecular exploitation, there had been no clinical trial to investigate the role and effects of 5α-reductase in prostate cancer in humans (15). The PCPT and REDUCE trials were designed to investigate the effect of finasteride (PCPT) and dutasteride (REDUCE) on the incidence of prostate cancer. Despite polarizing designs and differences in the composition of patient populations enrolled in the studies, the overall results and conclusions were analogous, simultaneously raising hope and concern for the use of 5α-RIs in the treatment of prostate cancer. This review aims to evaluate these two landmark clinical trials within the framework of the current debate, and reviews pertinent studies regarding the use of targeting of 5α-reductase for prostate cancer chemoprevention through October 2013.

## 2. Trials of prevention, PCPT vs. REDUCE: Prostate cancer finds its ‘groove’

Data from the PCPT were published in July 2003 in the New England Journal of Medicine (16). This study tested whether lowering circulating androgen levels in the prostate could reduce the risk of developing prostate cancer. Inclusion criteria were males aged ≥55 years old with PSA levels ≤3.0 ng/ml and a normal digital rectal examination (DRE) result. Patients who were randomized had annual PSA/DRE tests, semi-annual clinic visits and quarterly telephone follow-up calls. A prostate biopsy was recommended for those exhibiting a PSA >4.0 ng/ml or abnormal DRE, and the majority of biopsies performed were 6-core biopsies. An adjustment factor of 2.3 was applied to the treatment arm to account for the biological impact of finasteride on lowering the serum PSA values, and to maintain an equivalent number of biopsies in the placebo group. The study, originally planned for a 7-year duration, was terminated 15 months early due to meeting the objectives of the study ahead of schedule. A significant decrease in the prevalence of prostate cancer in the finasteride-treated group, as compared with the placebo group (18.4 vs 24.4%, P≤0.001), was observed. The impact of these data was reduced due to the observations that of the patients diagnosed with prostate cancer, an increased number of patients were found to have high-grade tumors (Gleason 7–10) in the finasteride-treatment group, as compared with the placebo group (37 and 22% respectively, P<0.001). Subsequently, this led to speculation among patients, urologists, epidemiologists and health policy makers as to whether or not finasteride, previously presented as an attractive chemoprevention agent for prostate cancer, was predisposing patients to a more aggressive disease, thus compromising treatment outcomes upon prostate cancer diagnosis.

The REDUCE trial was designed to recruit men at an increased risk for prostate cancer with no evidence of the disease at baseline (17). Initial results of the REDUCE trial were published in the New England Journal of Medicine in April 2010 with great anticipation of the radical effect on blocking 5α-reductase action upon the initiation of malignant prostate growth (18). The study was planned for 4 years of follow-up, and protocol biopsies were planned at 2 and 4 years in efforts to minimize the ‘for cause’ biopsies. These biopsies were performed at the discretion of the clinician, likely based on elevated PSA levels or an abnormal DRE, but this was not explicitly defined in the study. The biopsies were obtained on an as-needed basis, between the 2 and 4 year scheduled biopsies. Biopsies were to be 10 cores to balance between the need to maximize the detection of clinically significant tumors, yet minimize the over-diagnosis of low grade tumors (17).

The primary endpoint of this study was the diagnosis of prostate cancer by a TRUS biopsy during the study. This endpoint was examined in several subgroups: Age, family history of prostate cancer, International Prostate Symptom Score, prostate volume, PSA level and body mass index. The incidence of prostate cancer was lower in the dutasteride arm in every subgroup. During the 4-year period of the trial, prostate cancer was diagnosed in 659 (19.9%) patients of the 3,305 patients in the dutasteride arm of the study and 858 (25.1%) of the 3,424 patients in the placebo arm, resulting in an absolute risk reduction of 5.1% and a relative risk reduction of 22.8%. The majority of tumors were low grade, with 70% of the total number of cancers exhibiting a Gleason score of 5 or 6. The number of tumors with Gleason grades of 7–10 did not differ significantly over the course of the 4-year study, or within the subgroup of biopsies taken at the 1- to 2- or 3- to 4-year point. The promise of an absence of a higher number of tumors with Gleason grade 8–10 at the end of 1–2 years in the dutasteride group, was counteracted by a significant difference detected in the number of tumors with Gleason grade 8–10 in the later follow-up (years 3–4) (12 and 1, dutasteride vs. the placebo group, respectively, P<0.003). This last finding from the REDUCE trial likely precipitated the denial by the Food and Drug Administration for the use of 5α-RIs as prostate cancer chemopreventative agents, serving to reiterate the controversial and apparently analogous results from the PCPT.

## 3. Discussion

The results of PCPT and REDUCE showed a significant reduction in the overall incidence of prostate cancer in patients administered with 5α-RIs, but there was an increased incidence of high grade tumors in the treatment arm of both studies. Numerous reports have aimed to dissect these two studies and debate whether or not the results reflect a true increase in incidence of high-grade tumors or if this is merely the result of an inherent bias within the studies. Debates have revolved around the number of patients excluded from the final analysis of the PCPT, increased sensitivity and specificity of prostate cancer detection in biopsies on patients in the treatment arms of each study and inherent selection biases in both studies.

The fact that a large number of patients in the PCPT were not included in the final analysis for various reasons requires serious consideration. In the finasteride group, 3,290/8,137 (46.3%) patients were not included in the final analysis due to death (7.0%), declining end of study biopsy (25.4%), being lost to follow-up (8.0%) or being excluded from the final analysis (5.8%). A similar number of patients were not included in the placebo group, with 3,016/8,158 (42.4%) patients excluded due to death (6.7%), declining end of study biopsy (22.8%), being lost to follow-up (7.4%) or being excluded from the final analysis (5.5%). Unanswered questions remain, with >40% of the data in each group not being included in the final analysis. Redman *et al* (19) created a statistical model to account for the large number of PCPT patients that did not have an endpoint (19). Covariates, such age, family history and treatment arm assignment were used to predict the outcomes of these patients (lacking reported outcomes), and such analysis revealed that prostate cancer prevalence remained significantly lower in the finasteride group as compared with the placebo (14.7 vs 21.1%, P<0.0001). There was no significant increase, however, in the high-grade cancer detected in the finasteride group as compared with the placebo (4.8 vs 4.2%, P=0.012).

Another potential bias in these studies is the increased sensitivity and specificity of the prostate biopsy in the treatment arms of each study. 5α-RIs are known to decrease prostate volume (20) and outcomes of the REDUCE trial documented a significant reduction in the prostate volume in the treatment arms as compared with controls, with the prostate volume in the placebo group of the REDUCE trial increasing from a mean of 45.8 ml at baseline to 52.3 and 56.2 ml at 2 and 4 years, respectively. The dutasteride group decreased from 45.7 to 38.6 ml, from baseline to 2 years, and remained stable at 39.0 ml at the 4-year mark as expected. Pinsky *et al* (21) aimed to estimate how this difference in volume would impact the sensitivity and specificity of prostate biopsy. Towards resolving this issue, a model was created to extrapolate the pathological findings from radical prostatectomy specimens and their correlation with biopsy specimens, to those who did not undergo a prostatectomy, but had been diagnosed with prostate cancer on biopsy. The misclassification rate (the rate of tumors that were low-grade on biopsy but high-grade on prostatectomy specimen) was significantly lower in the finasteride arm (34.6%) as compared with the placebo (52.6%). Given the low overall number of high-grade tumors, this could be underlying the observed increased sensitivity for detecting high-grade disease on biopsy in the finasteride arm and a false increase in true high-grade cancers in the finasteride group.

Cohen *et al* (22) also investigated the effect of prostate volume on high-grade disease detection. Development of a logistic regression model based on prostate volume as well as race, family history, age and number of biopsy cores, indicated that the association of gland volume and detection of high-grade cancer existed independently of the drug effect. A second model designed to predict an odds ratio for low- and high-grade disease, revealed no difference in the high-grade disease between the finasteride and placebo groups after volume adjustment. Similarly, Kaplan *et al* (23) created a logistic regression model that adjusted the results based on the sampling density of biopsies in the PCPT. They showed that finasteride significantly reduced the risk of prostate cancer relative to the placebo, across multiple groups of prostate cancer (Gl 4–7), with no significant effect on Gl 2–3 or 8–10 prostate cancer groups (23). Another model integrating the varying sensitivity of prostate biopsies and its potential effect on prostate cancer detection (19), revealed a risk of high-grade disease either equivalent to or less than the placebo in the finasteride arm. Biopsy sensitivity was shown to have to be >85% in the placebo group and 25–30% in the finasteride group for there to be a significant increase in high-grade disease in the finasteride group, based on these models. This combination seems highly unlikely given that finasteride can increase the sensitivity for detection of high-grade tumors. This evidence implicates inherent biases in the PCPT trial design being responsible for the observed increase in high-grade tumors in the treatment arm, thus specifically illustrating how small changes in biopsy sensitivity could explain observed increased risk of high-grade prostate cancer.

Recognition of the role of selection bias in the PCPT trial undermining the selection of patients for prostate biopsy may provide a constructive platform. Despite the attempt to have an even number of biopsies in each group by adjusting the PSA in the treatment arm by 2.3 (vs. 2), recommendations for biopsy were higher in the placebo group (24.8%) as compared with finasteride treatment (22.5%). A significantly lower number of men (P<0.001) underwent a biopsy for elevated PSA in the finasteride group (48.4%) as compared with the placebo (56.5%). This investigator-generated bias could distinctly impact the PCTP outcome on two tiers. Firstly, the correctional factor of 2.3 may have led to a disproportionate decrease in the number of biopsy recommendations in the finasteride treatment group and delayed the diagnosis of prostate cancer. Secondly, an increased proportion of biopsies, prompted by an abnormal digital rectal examination, may have led to an increased number of diagnoses of high-grade cancers as previously established (24). In retrospect, it may have been more useful to use the correction factor of 2 for serum PSA levels in the finasteride-treated patients. This would have more closely simulated clinical practice while potentially avoiding the aforementioned selection biases.

There was potential selection bias in the REDUCE trial. A higher number of patients were diagnosed with prostate cancer at the 1–2 year mark in the placebo group and were therefore removed from the study. A proportion of these patients with initial low-grade (Gl 5–6) tumors may have progressed to higher grade (Gl 7–10) if left in the study and re-biopsied. One smaller study showed a significant number of tumors that increased in grade over a similar time interval (25). Should these results provide a direct translation into the REDUCE trial, it is unlikely that a significant increase in the number of high-grade tumors would have been found in the treatment arm.

Both PCPT and REDUCE used prostate cancer incidence as a primary endpoint. One recent study used data from the Prostate, Lung, Colorectal and Ovarian trial to predict prostate cancer mortality using prostate cancer incidence data from the PCPT and REDUCE trials (26). Prognostic data, such as Gleason score, PSA and clinical staging from each study were used, as were aforementioned adjusted results from PCPT based on increased sensitivity of prostate biopsy from a decreased prostate volume. These results showed no significant differences in prostate cancer mortality in these studies, with a trend towards an increased risk using the original PCPT date, and a trend towards decreased risk using the adjusted PCPT and REDUCE data. These data were corroborated by a more recent updated analysis of patients from the PCPT, which showed no significant differences in overall survival between the placebo and treatment groups (27).

Perhaps the most compelling evidence that the observed increased incidence of high-grade prostate cancer is unrealistic stems from a study estimating the number of high-grade tumors if all the patients with a positive biopsy had undergone a prostatectomy (19). Data from 500 patients who underwent a prostatectomy of the 2,017 patients diagnosed with prostate cancer in PCPT were analyzed, towards a model development to evaluate factors increasing the odds of undergoing a prostatectomy and subsequent risk of these patients having high-grade disease. Issues of selection bias become quickly apparent, as noted by the authors; the patients who underwent a prostatectomy were not a random sample. A younger age, PSA at randomization and biopsy prompted by PSA or DRE were all positive predictors of a prostatectomy. These patients were also more likely to be diagnosed with prostate cancer on ‘for cause’ biopsies with a longer time observed after diagnosis. Upon factoring in this bias, the adjusted results of the PCPT are encouraging. The estimation of prostate cancer prevalence showed a decreased risk of overall and high-grade disease in the finasteride arm as compared with the placebo, resulting in a 27% relative risk reduction for high-grade tumors (95% confidence interval, 0.56–0.96; P=0.02) (19).

## 4. Future REDEEM-ing of 5α-reductase inhibition

It is important to determine where these discussed considerations may lead. Both the PCPT and REDUCE trials unfolded a provocative data in the analysis of prostate cancer chemopreventative strategies. Table I summarizes key similarities and differences between the two trials. The PCPT was a larger study that was comprised of patients with much lower baseline PSA and normal DRE, and no previous biopsies. The results revealed a decreased number of patients diagnosed with prostate cancer in the finasteride group. Of those patients, however, there was a significant increase in the number of higher-grade tumors as compared with the placebo. The REDUCE trial was designed to evaluate patients with an increased risk of prostate cancer, based on elevated PSA, and with recent prior negative prostate biopsy. The majority of biopsies (93%) were scheduled accordingly to eliminate clinician-generated biases, based on rectal examination and PSA. Mirroring the outcomes of the PCPT trial, REDUCE also revealed a significant decrease in prostate cancer incidence in the treatment arm, and an increased number of high-grade tumors following treatment.

The significant decrease in prostate cancer prevalence in both the PCPT and REDUCE trials raises hope for potential chemoprevention agents in the fight against prostate cancer. This emerges as an increasingly important argument with the changing landscape of medicine and the intense scrutiny over the diagnosis and treatment of prostate cancer. The acceptance for the use of 5α-RIs in prostate cancer chemoprevention is the notion of their potential effect in predisposing patients to high-grade prostate cancer. This is an unfortunate consequence from the over-interpretation of findings from the PCPT and REDUCE trials. Numerous mathematical models reflect with confidence that 5α-RIs are not likely to increase risk for high-grade prostate cancer, and significantly enough others predict an actual reduced risk for all grades of prostate cancer by these agents (19,21–23). Furthermore, follow-up data from the REDUCE trial following an additional 2 years, showed no new Gleason 8–10 tumors (28). Despite the strong collective evidence towards a chemoprevention action that can be clinically exploited, support has not been sufficiently powerful for the Food and Drug Administration’s Oncologic Drug Advisory Committee to approve the use of 5α-RIs for prostate cancer chemoprevention (29).

Besides the potential for chemoprevention, the therapeutic value of 5α-RIs has also been interrogated in the treatment of prostate cancer during the progression to an advanced disease. The REDEEM trial revealed great promise that dutasteride may decrease the progression of low-grade prostate cancer in men undergoing active surveillance (30). Additional ongoing trials are pursuing the potential therapeutic value of 5α-RIs in patients facing biochemical failure following definitive therapy and adjuvant therapy with bicalutamide in castration-resistant prostate cancer. One could easily argue that there is a subset of patients who would benefit from 5α-RI treatment, depending on the cellular landscape of individual tumors, but this subset requires stringent profiling, without factoring in cost-effectiveness. Kattan *et al* (31) reported that dutasteride may be cost-effective as a chemoprevention agent, but only in patients at high-risk for prostate cancer diagnosis. Reports by an independent investigative team, however, sharply challenged the cost-effectiveness of finasteride, based on survival and quality of life as outcomes of success (32). As controversy still surrounds the value of the 5α-RIs in impairing prostate cancer, additional studies are warranted to determine a safe, efficacious, cost-effective chemopreventive agent to be given to young men, for preventing the disease in the ageing population. The non-biased counseling of patients on the risks and benefits associated with the 5α-reductase inhibitors and their potential for chemoprevention of prostate cancer is built on the clinical impact of the two trials, PCTP and REDUCE.

## Figures and Tables

**Table I:** Design comparison of the PCPT and the REDUCE trial.

	PCPT	REDUCE
Publication date	7/2003	4/2010
Designed length of study (years)	7	4
5α-reductase inhibitor	Finasteride (Proscar)	Dutasteride (Avodart)
Cancer risk in cohort	Low risk (PSA <3, normal DRE)	Higher risk (PSA 2.5–10)
Biopsy prior to study	No	Yes
Number enrolled	24,482	8,231
Number randomized	18,882	8,122
Number in final analysis	9060	6,706
PSA correction factor	2.3	2
Decision for biopsy	PSA >4, abnormal DRE and at end of study	Protocol at 2 and 4 years
Median number of biopsy cores	6	10
Overall incidence of prostate cancer (%)	21.5	22.5
Overall relative risk reduction (%)	24.8	22.8
Concern for increased risk of HG tumors	6.4% of patients with Gl 7–10 in treatment arm compared to 5.1% in placebo arm	12 Gl 8–10 tumors in treatment arm in years 3–4 compared to only 1 in placebo arm in years 3–4

PCPT, Prostate Cancer Prevention Trial; REDUCE, Reduction by Dutasteride of Prostate Cancer Events Trial; PSA, prostate-specific antigen; DRE, digital rectal examination; HG, high grade; Gl, Gleason score.

## Abbreviations

PCPT: Prostate Cancer Prevention Trial
REDUCE: Reduction by Dutasteride of Prostate Cancer Events
PSA: prostate-specific antigen
DRE: digital rectal examination
